# Preliminary Study on Selected Markers of Oxidative Stress, Inflammation and Angiogenesis in Patients with Bladder Cancer

**DOI:** 10.1007/s12253-019-00620-5

**Published:** 2019-03-04

**Authors:** Ewa Sawicka, Ewa Maria Kratz, Beata Szymańska, Anna Guzik, Artur Wesołowski, Paweł Kowal, Lilla Pawlik-Sobecka, Agnieszka Piwowar

**Affiliations:** 1 Department of Toxicology, Faculty of Pharmacy with Division of Laboratory Diagnostics, Wroclaw Medical University, Borowska Street 211, 50-556 Wrocław, Poland; 2 Department of Laboratory Diagnostics, Faculty of Pharmacy with Division of Laboratory Diagnostics, Wroclaw Medical University, Borowska Street 211a, 50-556 Wrocław, Poland; 3 Students Scientific Society at the Department of Toxicology, Faculty of Pharmacy with Division of Laboratory Diagnostics, Wroclaw Medical University, Borowska Street 211, 50-556 Wrocław, Poland; 4 Department and Clinic of Urology and Urological Oncology, Faculty of Postgraduate Medical Training, Wroclaw Medical University, H. M. Kamieńskiego Street 73a, 51-124 Wrocław, Poland

**Keywords:** Bladder cancer, Cancer grade and stage, Oxidative stress, Inflammation, Angiogenesis

## Abstract

In recent years, bladder cancer (BC) has been reported as one of the most commonly occurring cancers among older people, and its detection is still difficult. Therefore, there is a need to search for additional useful markers of disease. Some studies indicate the important roles of inflammation and oxidative stress (OS) in bladder tumour pathogenesis. The aim of this study was to examine the levels of selected markers of OS, inflammation and angiogenesis in blood plasma/serum samples derived from patients with BC, and a healthy control group. Moreover the degrees of change and strength of correlation between values of the analysed markers and tumour stage or grade were estimated. Concentrations of: malondialdehyde (MDA) and advanced oxidation protein products (AOPP), and total antioxidant status (TAS) divided into slow (TAS-s) and fast (TAS-f) antioxidants (spectrophotometric measurement), angiogenin (ANG) (immunoenzymatic method) and C-reactive protein (CRP) (immunoturbidimetric method) were determined in both the studied groups. The majority of values of the examined parameters were significantly higher among patients, while subfractions of TAS were significantly lower in comparison to the control group. Moreover, different values and different strengths of correlation between the examined parameters and cancer stage or grade were noticed. The most significant changes for CRP were observed in T2 and for MDA in G3, while the lowest TAS-f activity was revealed in G1 patients. Increased values of OS parameters, angiogenesis and inflammation markers, in combination with reduced TAS subfractions activity in BC are important in its pathogenesis and will be helpful in estimation of patients’ condition.

## Introduction

In recent years bladder cancer (BC) has been reported as one of the most commonly diagnosed cancers among people aged over 65 years. Estimated data presented by the World Health Organization (WHO) in 2012 showed that almost 7% of new oncological diseases of the bladder are tumours [1–3]. Recently, there has been increased interest in studies of the role of free radicals in carcinogenesis and the role of antioxidants in the prevention and treatment of cancers [4, 5]. Oxidative stress (OS) promotes cell proliferation in vitro, with both superoxide and hydrogen peroxide stimulating growth [6]. Inherent oxidative stress may change certain functions in cancer cells and tissues (cell proliferation, promotion of mutations, invasion and metastasis) [7, 8]. Information about the role of oxidative stress in BC is still incomplete, but some scientific data indicate the involvement of OS in the formation and development of bladder cancer. Redox disorders are characteristic for both the initiation and progression of BC. In addition, many studies indicate a role of OS in the regulation of MAPK cascade (mitogen-activated protein kinases) and its involvement in carcinogenesis and metastasis consisting BC. Examples of kinases belonging to the MAPK family are ERK kinases (extracellular signal-regulated kinases), whose expression is proportional to the severity and malignant of BC [9–11].

Oxidative damage plays a significant role in the pathogenesis of many non-cancer diseases. Free radical mechanisms have been implicated in the pathogenesis of e.g. atherosclerosis, ischemic heart disease, diabetes, rheumatoid arthritis and neurodegenerative diseases [12]. Many authors have documented that an increased level of lipid peroxidation products are also related to cancer development [13, 14]. Recent single studies have shown that intensive lipid peroxidation appears in the tissues of patients with BC, and its product – malondialdehyde (MDA) can form adducts with various macromolecules, influencing cell proliferation [15]. Proteins are also exposed to OS. Advanced oxidation protein products **(**AOPP) are formed in the human organism in the progression of many diseases, mainly from albumins and methionine- or tyrosine-rich polypeptides by the oxidative attack of a reactive oxygen species. Diagnostic or prognostic utility of AOPP are especially indicated in the course of diabetes, rheumatoid arthritis, development of pregnancy complication both in mother and child, and dementia. However, AOPP measurement seems to be most promising in blood plasma or urine in the course of some cancer diseases: large intestine and stomach, as well as thyroid gland [16, 17]. There is no literature data on AOPP changes in patients with BC.

It is already known that the adverse effects of free radicalsprocesses are controlled by the antioxidant system present in the human organism. However, this system works properly only if balance exists between its components [18]. The antioxidant system consists of diverse substances and enzymes. Total antioxidant status (TAS) can be considered as two separate values: 1) TAS-s for slow-acting enzymatic antioxidants, such as glutathione peroxidase (GPx), superoxide dismutase (SOD) and other enzymes, and 2) TAS-f for non-enzymatic fast-acting antioxidants, like e.g. β-carotene, tocopherols, bioflavonoids, ascorbic acid, glutathione and albumin [19, 20].

Oxidative stress plays an important role in BC progression, it can also participate in angiogenesis of the tumour. Angiogenesis - the formation of new capillaries from pre-existing blood vessels, is vital for tumour growth and metastasis [21–24]. Angiogenin (ANG), as one of the elements of this process, is polypeptide with the ability to induce new blood vessel growth. ANG have a significant role in the angiogenesis of cancer causing migration, proliferation and differentiation of endothelial cells [25, 26]. Accumulating evidence suggests that the expression and activity of ANG increased significantly in a variety of human cancers. Shu et al. [27] proved for the first time that ANG could play a pivotal role in the development of bladder cancer through regulating AKT/mTOR (serine threonine kinase/mammalian target of rapamycin) signaling pathway. The authors indicate that ANG could be a good diagnostic and therapeutic target for controlling bladder cancer process [27].

Apart from oxidative stress and angiogenesis, inflammation may also be important in the pathogenesis of BC especially in the aspect of tumour microenvironment and carcinogenesis [28–30]. Despite the fact that chronic inflammation is characteristic for many non-cancer diseases, for example: inflammatory bowel diseases, rheumatoid arthritis, neurodegenerative diseases, diabetes, asthma or obesity, it causes oxidative stress which can trigger the process of carcinogenesis through changes in molecules and signal paths, and partial destruction of tissue homeostasis [31–33]. The observational and experimental studies confirm the participation of inflammatory processes in cancer. The best-documented examples include cancer intestine caused by chronic inflammatory disease (ulcerative colitis thick or Leśniowski-Crohn’s disease), hepatocellular carcinoma related with viral hepatitis, as well as squamous bladder cancer urinary tract infection as a consequence of infection with blood clots [34, 35].

The aim of this study was to investigate the changes of values and analysis of differences between the determined parameters of oxidative stress (TAS-s and TAS-f, MDA, AOPP), angiogenesis (ANG) and inflammation (CRP) in blood plasma/serum of patients with bladder cancer and the control group. Additionally, we searched for an association between levels of the estimated markers and stages or grades of BC.

## Material

The study was conducted in a group consisting of 45 patients with diagnosed BC in different stages of clinical advancement. They were hospitalized at the Department and Clinic of Urology and Urological Oncology, Wroclaw Medical University. The grade and stage of tumours were determined according to the TNM (Tumor Nodules Metastases) Classification of Malignant Tumours [36] on the basis of histopathological examination. The control group was composed of 20 healthy volunteers with no history of cancer or chronic inflammation. The BC patients and subjects from the control group were of similar socioeconomic status. Demographic and clinical data for the BC patients and the control group are shown in Table 1.

**Table 1:** Characteristic of bladder cancer patients and control group

Features	N (%)
Patients	45
Male	37 (82)
Female	8 (18)
Age in years (median) - male	72
Age in years (median) - female	69
Clinical stage	
Ta	21 (50.0)
T1	15 (35.7)
T2	6 (14.3)
Clinical grade	
G1	16 (35.5)
G2	23 (51.1)
G3	6 (13.3)
Controls	20
Male	9 (45)
Female	11 (55)
Age in years (median) - male	69
Age in years (median) - female	66

Ta = non-invasive papillary tumour; T1 = tumours invading sub epithelial connective tissue; T2 = tumours invading the muscle of bladder wall; G1 = low grade, G2 = moderately grade, G3 = high grade; N = number of cases, % = percentage of cases

Whole human blood (3 ml) was collected from both groups (patients and control) in fasting state. In order to obtain plasma or serum, the blood sample was placed into tubes with or without anticoagulant agent (3.2% buffered sodium citrate), respectively. Then both types of tubes were centrifuged at 1480×g for 10 min to separate the plasma or serum, respectively. The material was kept at −80 °C until analysis. All samples were collected after obtaining informed consent from all individuals, and the study was approved by the Wroclaw Medical University Ethics Committee (KB-13/2014 and KB-276/2016).

## Methods

### MDA

Concentration of MDA in serum samples was determined by a method invented by Yoshioka et al. [37]. The serum sample was incubated in a boiling water bath with 0.67% thiobarbituric acid solution (TBA, Sigma Aldrich) to obtain a pink chromogen ([TBA]_2_-malondialdehyde adduct). The resulting chromogen absorbance was determined at the wavelength of 535 nm. The concentration of MDA was read from a standard calibration curve plotted using 1,1,3,3′-tetramethoxy propane (TEP, Sigma Aldrich). The spectrophotometric measurement was carried out using Hitachi U-2900 spectrophotometer.

### AOPP

Determination of AOPP in the plasma samples was conducted by the spectrophotometric method described by Witko-Sarsat et al. [16]. Plasma samples were treated with 1.16 M potassium iodide (Sigma Aldrich) and 10% acetic acid (Merck Millipore), then mixed and immediately measured at 340 nm against a blank reference (0.9% NaCl). The concentration of AOPP was read from a standard calibration curve plotted using 100 μM Chloramine T (Sigma Aldrich). The intensity of the measured absorbance is directly proportional to its concentration in the sample. The spectrophotometric measurement was carried out using a Hitachi U-2900 spectrophotometer.

### TAS (TAS-s and TAS-f)

To estimate the value of TAS for fast and slow plasma antioxidants (TAS-f and TAS-s respectively) we used the Re et al. method [38]. The method is based on measurements of ABTS^+^ (monocation of 2,2′-azino-bis-(3-ethylbenzothiazoline-6-sulfonic acid; Sigma Aldrich) reduction by antioxidant substances contained in the plasma. ABTS^+^was obtained by the reaction of potassium persulfate with ABTS (2,2′-azino-bis-(3-ethylbenzothiazoline-6-sulfonic acid; Sigma Aldrich). ABTS^+^ solution is blue-green, while its reduced form is colourless. Each sample was measured twice at 414 nm: at first immediately after adding plasma into a probe, and then after 5 min to obtain the delta of absorbance. The degree of reduction is proportional to the activity and concentration of antioxidants in the plasma. The level of antioxidants was read from a standard calibration curve plotted using Trolox and the appropriate mathematical formula to calculate TAS-s and TAS-f activity.

### ANG

To determine the concentration of ANG in plasma the commercial enzyme immunoassay kit – ELISA (R&D System) was used. This assay employs the quantitative sandwich enzyme immunoassay technique using appropriate monoclonal and polyclonal antibodies specific for human ANG. The results were read with a microplate reader STAT FAX 2100® at 450 nm.

### CRP

For determination of CRP concentration in serum samples, the immunoturbidimetric method was used (DiaSys, Germany). The CRP reacts with the specific antibody producing insoluble immune complexes. The turbidity caused by these reactions is proportional to the CRP concentration in the sample and can be measured spectrophotometrically by the autoanalyzer Konelab™.

## Statistical Analysis

Statistical analysis was performed using Statistica PL software (version 12.0) for Windows. The normality of distribution was checked with the Lilliefors test. Student’s t test for parametric data and the Mann-Whitney U test for nonparametric data were used for the appropriate variables. The correlations between study results were analyzed by the Spearman and/or Pearson tests (nonparametric and parametric test, respectively). In all analyses *p* < 0.05 was accepted as a significant value.

## Results

The levels of the examined parameters: MDA, AOPP, ANG, CRP, TAS-s and TAS-f in patients and control group are presented in Table 2. We observed that the values of almost all the examined parameters, except CRP, were significantly different among patients with BC in comparison to the control group. Although CRP concentration has the most growth among the mentioned parameters, we did not observe any significant differences between the analysed groups. Significantly higher values were noticed for MDA, AOPP and ANG (about 1.3-, 2.3- and 1.5-times, respectively) in the studied group than in the control. The opposite effect was observed for the activity of TAS-f and TAS-s, which was significantly lower (about 0.9-times) in patients with BC in comparison to the control.

**Table 2:** The concentration of MDA, AOPP, ANG, CRP, TAS-f and TAS-s activity in bladder cancer patients and control group

Marker	Patients’ group (*N* = 45)		Control group (*N* = 20)		*p*
	Median (range)	Mean ± SD	Median (range)	Mean ± SD	
MDA [nmol/mL]	6.50 (4.10–9.50)	6.55 ± 1.51	5.10 (3.00–7.10)	5.14 ± 1.24	0.0005*
AOPP [μmol/L]	221.90 (65.90–1515.40)	279.96 ± 246.86	102.55 (62.90–249.60)	119.26 ± 53.24	0.0056*
TAS-f [μmol TE/L]	10.80 (1.70–16.70)	10.76 ± 2.13	11.85 (10.20–16.30)	12.05 ± 1.43	0.0167*
TAS-s [μmol TE/L]	15.10 (9.70–18.30)	14.95 ± 1.51	15.95 (13.50–19.00)	16.15 ± 1.55	0.0048*
ANG [ng/mL]	276.20 (102.30–512.40)	270.90 ± 111.61	184.85 (27.30–294.00)	181.72 ± 54.61	0.0012*
CRP [mg/mL]	3.20 (1.10–110.50)	12.79 ± 27.54	3.50 (3.00–1.30)	4.86 ± 3.23	0.2058

*SD* standard deviation; *p* - value of differences between patients’ and control group; *significant difference between groups; MDA malondialdehyde; *AOPP* advanced oxidation protein products; *ANG* angiogenin; *CRP* C-reactive protein; *TAS-f* and *TAS-s* fast and slow antioxidants, respectively; *TE* Trolox Equivalent; *N* number of cases

**Fig. 1:**
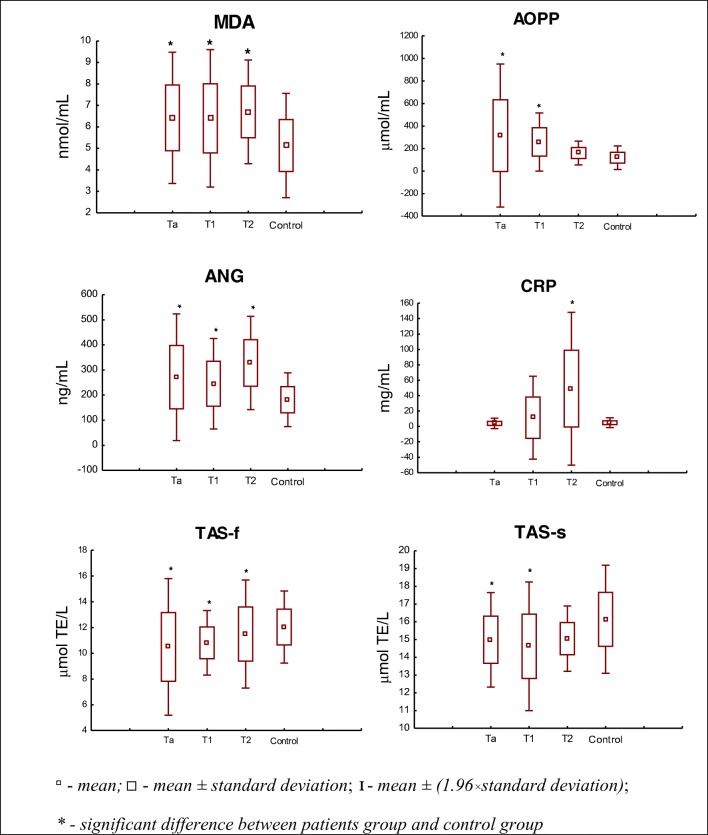
Levels of examined parameters: MDA, AOPP, ANG, CRP, TAS-f and TAS-s in BC subgroups relating to the tumour stage (T), in comparison to the control group

The levels of MDA, AOPP, ANG, CRP, TAS-f and TAS-s in patients with different stages of bladder cancer are shown in Fig. 1. As we observed, the levels of the majority of estimated parameters were significantly higher in different stages of cancer (Ta, T1, T2) in comparison to the control group. Only parameters of antioxidative defence (TAS-f and TAS-s) demonstrated significantly lower values in the BC subjects than in the control.

The concentrations of MDA in serum were significantly higher for patients in all tumour stages, especially at Ta (*p* = 0.0058), when compared to the control group (Table 3, Fig. 1). In more advanced stages of carcinoma the mean values of MDA were slightly lower than at Ta, but they were still significantly elevated in comparison to the control: T1 (*p* = 0.0135), T2 (*p* = 0.0119) (Table 3, Fig. 1). No differences were observed in MDA median concentration between Ta, T1 and T2 stages (Table 3).

**Table 3:** The concentration of MDA, AOPP, ANG, CRP, TAS-f and TAS-s activity in the subgroups of bladder cancer stage (T)

Marker	Ta	T1	T2
	Median (range)		
MDA [nmol/mL]	6.6 (4.1–9.1)	6.4 (4.3–9.5)	6.4 (5.3–8.6)
AOPP [μmol/L]	252.0 (67.9–1515.4)	258.1 (65.9–500.4)	151.9 (92.3–258.5)
TAS-f [μmol TE/L]	10.6 (1.7–16.7)	10.7 (7.9–13.0)	11.6 (8.3–14.0)
TAS-s [μmol TE/L]	15.0 (11.6–18.3)	15.2 (9.7–16.6)	15.2 (13.9–16.4)
ANG [ng/mL]	276.2 (102.9–512.4)	247.6 (102.3–382.2)	356.1 (153.6–429.4)
CRP [mg/mL]	3.0 (1.1–13.9)	3.2 (2.0–110.5)	44.7 (1.6–100.4)

*Ta*, *T1*, *T2* appropriate cancer stage; *MDA* malondialdehyde; *AOPP* advanced oxidation protein products; *ANG* angiogenin; *CRP* C-reactive protein; *TAS-f* and *TAS-s* fast and slow antioxidants, respectively; *TE* Trolox Equivalent

The AOPP concentrations in plasma were the highest in T1 patients (*p* = 0.0001), and were about three times higher in comparison to the control group (Table 3, Fig. 1). Also for Ta (*p* = 0.0109), the AOPP level was significantly increased as compared with the control (Table 3, Fig. 1). However, the AOPP concentration was lower at T2 stage when compared with Ta and T1 stages (Table 3), but the differences do not reach the significant level.

Considering the marker of angiogenesis the highest concentrations of ANG were observed among patients with invasive bladder cancer at T2 (*p* = 0.0001) (Fig. 1). Almost all remaining types of bladder cancer according to stages Ta and T1 were also characterized by significantly higher values of ANG in comparison to the control (*p* = 0.0065, *p* = 0.0157), respectively (Fig.1). Additionally when the median value of ANG concentrations were analysed at Ta and T1, it can be concluded that they are similar, but lower than for T2 stage (Table 3).

The CRP concentration in serum that characterizes the intensity of inflammatory response, was significantly higher (*p* = 0.0004) only in one group of patients - with T2 stage, in comparison to the control (Fig. 1). In other types of cancer stages the differences in CRP values were not significant against the control (Fig. 1). However, at Ta and T1 stage of BC the CRP concentration was visibly lower than at T2 stage (Table 3).

The measurement of TAS activity in the plasma of patients with BC demonstrated a significant decrease in antioxidant activity, both for TAS-f and TAS-s (Fig. 1). The highest significant decrease of TAS-f was noticed in all types of cancers: Ta, T1 and T2 in comparison to the control (*p* = 0.0287, *p* = 0.0131, *p* = 0.0137, respectively) (Fig. 1). The TAS-s was significantly lower in cancer at Ta and T1 when compared to the control (*p* = 0.0153, *p* = 0.0132, respectively) (Fig. 1). The activities of TAS-f and TAS-s were on a similar level when their median values were compared between Ta, T1 and T2 stages of BC (Table 3).

Moreover, we also observed the presence of certain linear correlations between levels of the examined parameters in BC patients. Firstly, a moderate negative correlation was noted between ANG concentration and the activity of TAS-s in the total studied group of BC patients (r = −0.352; *p* = 0.0287). Additionally, moderate and very strong negative correlations between these parameters were observed at T1 (r = −0.5432, *p* = 0.036) and T2 (r = −0.8513, *p* = 0.032), respectively. Moreover, at T2 a very strong positive correlation between ANG and TAS-f (r = 0.8465, *p* = 0.034) was also observed. Additionally we noticed a strong positive correlation at T1 between TAS-f and MDA (r = 0.6568, *p* = 0.009).

**Fig. 2:**
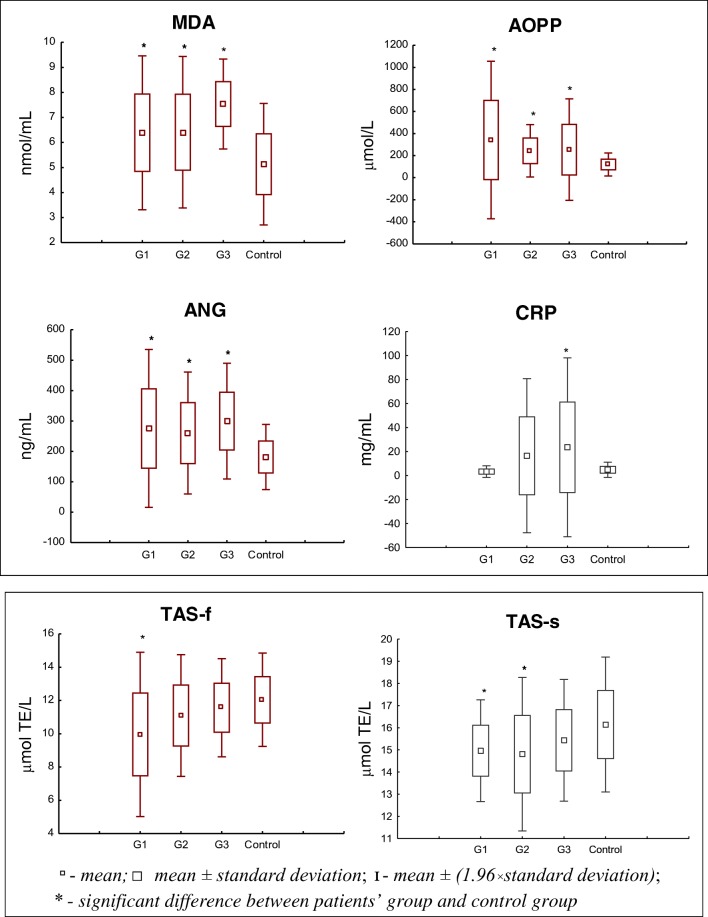
The values of examined parameters: MDA, AOPP, ANG, CRP, TAS-f and TAS-s in BC subgroups relating to the tumour grade (G) in comparison to the control group

We observed that the majority of parameters had significantly higher values in the clinical advancement of cancer (G1, G2, G3) in comparison to the control group. However, TAS activity (both subtractions) in plasma was significantly lower (G1, G2) than in the control. The values for all examined parameters are shown in Fig. 2.

In the case of MDA the highest value was revealed in subgroup G3 and it was significantly higher (about 40%) when compared to the control group (*p* = 0.0002) (Fig. 2, Table 4). In the other clinical advancements of cancer the levels of MDA were similar (Fig. 2). In G1 and G2 subgroups the MDA concentrations were also significantly elevated in comparison to the control (*p* = 0.0019, *p* = 0.0051, respectively) (Fig. 2). The median values of MDA concentrations were lower in G1 and G2 in BC grade than in the G3 (Table 4).

**Table 4:** The concentration of MDA, AOPP, ANG, CRP, TAS-f and TAS-s activity in subgroups of bladder cancer grade (G)

Marker	G1	G2	G3
	Median (range)		
MDA [nmol/mL]	6.7 (4.1–9.1)	6.4 (4.1–9.5)	7.7 (6.3–8.6)
AOPP [μmol/L]	252.2 (67.9–1515.4)	258.1 (65.9–500.4)	154.6 (92.3–720.5)
TAS-f [μmol TE/L]	10.6 (1.7–12.0)	10.7 (7.9–16.7)	11.4 (9.6–14.0)
TAS-s [μmol TE/L]	15.1 (11.6–17.7)	15.0 (9.7–18.3)	15.3 (13.9–17.7)
ANG [ng/mL]	252.6 (102.9–512.4)	276.2 (102.3–440.8)	265.3 (211.2–429.4)
CRP [mg/mL]	3.0 (1.1–20)	3.2 (0.4–110.5)	7.4 (2.7–100.1)

*G1*,*G2*,*G3* appropriate cancer grade; *MDA* malondialdehyde; *AOPP* advanced oxidation protein products; *ANG* angiogenin; *CRP* C-reactive protein; *TAS-f* and *TAS-s* - fast and slow antioxidants, respectively; *TE* Trolox Equivalent

The significantly highest concentration of AOPP (*p* = 0.0062) was observed in G1, G2 and G3 when compared to the control (Table 4, Fig. 2). The decreasing AOPP concentration together with the increasing grade of cancer (G1 and G2 versus G3) was also observed (Table 4).

The concentrations of ANG in BC were significantly higher in comparison to the control group in all subgroups of tumour grade (Fig. 2). We noticed the significantly highest ANG concentration particularly in G3 (about 1.7-time) in comparison to the control (*p* = 0.0008) (Fig. 2). In other subgroups of individuals the ANG values were similar but they were also significantly higher when compared with the healthy subjects (Fig. 2). No differences were noticed between median values of ANG concentrations when BC grades were compared (Table 4).

The CRP level increases in subsequent subgroups of cancer grade, but was significantly higher only for patients with G3 (*p* = 0.0268) in comparison to the control (Fig. 2). In the G2 group the CRP concentrations were also higher than in control subjects, however, the differences were not significant. A low CRP level, similar to those measured for the control, was noticed for G1 patients (Fig. 2). In median values of CRP concentrations no differences between G1 and G2 groups of BC were observed (Table 4).

The results of TAS activity in blood plasma has pointed to a significant decrease in comparison to the healthy volunteers for G1 and G2, whereas TAS-f activity was significantly lower in the G1 grade only (Fig. 2). In TAS-f we noticed slightly increased activity together with the clinical advancement of cancer in comparison to the control (Fig. 2). Concerning TAS-s in G1 and G2 subgroups we noticed similar median values of this parameter (Table 4), however, their levels were significantly lower in comparison to the control group (*p* = 0.034, *p* = 0.0124, respectively) (Fig. 2).

We also revealed the existence of some correlations between the determined parameters. We noticed positive moderate correlations in G2 between: MDA and ANG (r = 0.4371, *p* = 0.037), and MDA and TAS-f (r = 0.4547, *p* = 0.029). Moreover, positive strong and moderate correlations were observed between TAS-f and ANG (r = 0.6423, *p* = 0.001), as well as between TAS-s and ANG (r = − 0.4145, *p* = 0.049).

## Discussion

Bladder cancer is one of the most common oncological diseases, especially among men. There are plenty of factors that may contribute to its development, both endogenous and exogenous. The metabolism of several compounds, like tobacco smoke, heavy metals, pesticides or nitrosamines, leads under certain conditions to free radical formation. Those highly reactive molecules are neutralised by antioxidants present in the blood plasma. However, if the organism is exposed to large quantities or the prolonged action of those exogenous substances generating free radicals, important cell structures may react, causing serious malformations, that later may start an oncogenesis [10].

In our study we attempted to make measurements of the biochemical parameters that characterize oxidative stress, inflammation and angiogenesis, such as: TAS-f and TAS-s, MDA, AOPP, CRP and ANG, in patients with BC. Some of these parameters were previously analysed in other oncological studies, nevertheless their examination in patients with bladder cancer is rare. To our knowledge estimation of TAS divided into slow and fast antioxidants has not yet been examined in BC patients, and neither has AOPP concentration. Our research is the first attempt to evaluate such a research panel of markers in bladder cancer and indicates its potential usefulness in reflection on and evaluation of the cancer process. We found that the activity of plasma antioxidants in patients with bladder cancer is significantly reduced in comparison to the control group. The mean value of TAS-f activity in BC was about 11% lower compared to the control. The obtained results are similar to the results of Badjatia et al. [39] who also indicated a significant decrease in serum antioxidant status in patients with BC. The authors examined levels of vitamins C and E, the activity of antioxidant enzymes and total antioxidant activity. In each case, they observed a significant reduction in activity of the analyzed parameters both in low grade, high grade and muscle invasive bladder cancers. However, they did not use the slow and fast separation of antioxidants. The reduction of the antioxidant systems activity in BC indicates its exhaustion, and suggests that the supplementation of exogenous antioxidants, like vitamins, might be helpful in anti-tumour prevention. Such a conclusion was presented in a cohort study conducted by Nechuta et al. [40]. Among oncological patients that were treated with vitamin supplements, the authors have observed a reduction in risk of death by 18%, compared to people who were not provided with those specifics. There is no data on the distribution of TAS in the literature for fast and slow antioxidants in bladder cancer. Our results indicate that the largest decrease in both TAS parameters, TAS-f and TAS-s, appeared in the case of Ta compared to healthy subjects. However, we found no proportional dependence between the activity of TAS subfractions and the stage of cancer. Badjatia et al. [39] showed that depletion of antioxidant status is the highest in high-grade bladder cancer and muscle invasive tumors.

Free-radical damage to lipids was reflected by the concentration of MDA in serum. The results showed that in BC peroxidation processes are significantly intensified in comparison to healthy people. The mean value of this oxidative damage marker was significantly higher, about 25%, in BC patients when compared to the control. We noticed that the concentration of MDA in serum rose mainly in G3 of BC. Among Ta, T1 and T2 the MDA level increased against the control by a similar percentage, while the study of Yalcini et al. [19], conducted in patients with urothelial cancer, indicated the growth of MDA concentrations among G1 and G2 patients. Also the study of Gecit et al. [41] confirmed that in BC the serum level of MDA is significantly increased, about 1.85-times, in comparison to controls. In our study MDA concentration is 1.27 times higher than in the control. Formation of AOPP in the human body is strictly correlated with the reaction between free radicals, especially hydroxyl radicals, and amino acids. Such proteins are accumulated inside cells and tissues and increase the risk of organelles dysfunction, mainly mitochondria, lysosomes and the cytoplasmic membrane. These abnormalities lead to changes in cell functioning that may result in apoptosis or, in extreme cases, carcinogenesis [33]. The results of our study regarding AOPP measurements in the plasma of patients with BC showed that AOPP concentrations were significantly higher (2.35-times) than in the control. There are no literature data involving the determination of AOPP in patients with BC. The data presented by Kosova et al. [42] confirms that elevated levels of plasma AOPP appears in cancerous diseases. In our study the highest concentration of AOPP was observed at Ta, which is the non-invasive but highly malicious stage, as well as in G1. Moreover, among the examined parameters, only for AOPP was a similar trend in changes of concentration in subsequent stages and grades of BC observed.

In our study the mean value of CRP was about 2.6-times higher for BC patients compared to control, however, these values doesn’t reach significant differences. The highest mean concentration of CRP was observed in the group of patients with malignant tumours at stage T2 and grade G3. According to the study by Gakis et al. [43] conducted in patients with BC, an increased level of CRP (more than 5 mg/L) was present in more than 50% of examined patients. Cancer cells are also able to produce many proinflammatory cytokines like IL-1 and IL-6, which stimulate the formation of CRP in the liver [44, 45].

An additional important parameter regarding tumour progression, which we examined, was ANG. This protein is responsible for the formation of new blood vessels in the closeness of tumour tissue. In all the examined groups of BC patients the results were significantly higher (1.5-times) in comparison to the control. The highest concentration of ANG was observed in stage T2, when cancer invades the muscle tissue of the bladder, as well as in grade G2. It was about 1.8- and 1.5-times higher, respectively, in comparison to the control. We may conclude that the intensity of angiogenesis rises depending upon the development stage of BC. Bigger and more malignant carcinomas even accelerate this process, by producing vascular formation stimuli. Some researchers have observed the connection between intensification of angiogenesis and BC progression. Elevated serum, plasma or urine angiogenin levels have been found in patients with BC [22]. Eissa et al. [26] analyzed the supernatants of urine samples from a diverse cohort of 97 patients. The median urinary angiogenin levels in BC, benign urological disorders and healthy volunteer groups were: 802.7, 425 and 33 pg/mL, respectively. Moreover the study of Zhao et al. [46] demonstrated that the plasma levels of ANG were significantly higher in patients who had bladder carcinoma compared with healthy control, also in patients who had recurrent disease compared with patients who were without recurrence. Therefore, an elevated plasma level of ANG may serve as a novel predictor for the risk of bladder carcinoma. Urquidi et al. [47] confirmed the above-mentioned results in BC patients showing that urinary angiogenin level was higher (410.98 pg/mL) vs. control group (44.58 pg/mL). Urinary angiogenin had a respectable diagnostic capability: sensitivity of 67%, specificity of 68%, positive predictive value of 96% and negative predictive value of 74% [47]. Based on the presented studies and own research, it can be seen that the level of ANG can be a measure of angiogenic activity in bladder cancer.

We showed different degrees of correlation between the examined markers in subsequent stages and grades of BC. If we consider TAS-f, mainly created by β-carotene, tocopherols, bioflavonoids, ascorbic acid, glutathione and albumin, we observe positive correlations between TAS-f with both ANG and MDA. It shows mobilization of these types of antioxidant reserves with simultaneous intensification of angiogenesis or lipids peroxidation (T1 stage or T2). Interesting correlations were observed especially in grade G2, where increased levels of MDA or ANG were connected with the mobilisation of antioxidants type TAS-f. Our study clearly indicates that the antioxidative system has close associations with redox processes and angiogenesis in the development of BC. Some authors indicated that not only endogenous but also exogenous antioxidants have impact on angiogenesis and factors modulating this process, and can be used as normalization of neovascularization agents [48].

## Conclusion

Our preliminary study showed that increased concentrations of markers characterizing oxidative stress and the inflammatory response of the human organism, such as MDA, AOPP and CRP as well as markers of angiogenesis, such as ANG, combined with the decreased activity of endogenous antioxidants in the case of bladder cancer, are potentially important findings. In our opinion the results of the present study are promising and worthy of future investigation in a larger cohort of patients with bladder cancer.

## Abbreviations

ABTS: 2,2′-azino-bis-(3-ethylbenzothiazoline-6-sulfonic acid)
ABTS^+^: Monocation of 2,2′-azino-bis-(3-ethylbenzothiazoline-6-sulfonic acid)
AKT/mTOR: Serine threonine kinase/mammalian target of rapamycin
ANG: Angiogenin
AOPP: Advanced oxidation protein products
BC: Bladder cancer
CRP: C-reactive protein
ERK: Extracellular signal-regulated kinases
G: Tumour grade
GPx: Glutathione peroxidase
IL-8: Interleukin-8
MAPK: Mitogen-activated protein kinases
MDA: Malondialdehyde
MMPs: Matrix metalloproteinases
RNS: Reactive nitrogen species
ROS: Reactive oxygen species
rRNA: Regulating ribosomal RNA
SOD: Superoxide dismutase
T: Tumour stage
Ta: Non-invasive papillary tumour
T1: Tumours invading sub epithelial connective tissue
T2: Tumours invading the muscle of bladder wall
TAS: Total antioxidant status
TAS-s and TAS-f: Slow and fast antioxidants, respectively
TBA: Thiobarbituric acid
TE: Trolox
TEP: 1,1,3,3′-tetramethoxy propane
TNM: Tumor Nodules Metastases
VEGF: Vascular endothelial growth factor
WHO: World Health Organization
